# Current knowledge on feline injection-site sarcoma treatment

**DOI:** 10.1186/s13028-017-0315-y

**Published:** 2017-07-17

**Authors:** Katarzyna Zabielska-Koczywąs, Anna Wojtalewicz, Roman Lechowski

**Affiliations:** Department of Small Animal Diseases with Clinic, Faculty of Veterinary Medicine, Warsaw University of Life Sciences, Nowoursynowska 159c, 02-776 Warsaw, Poland

**Keywords:** Chemotherapy, Clean margin, Immunotherapy, Radical surgery, Radiotherapy

## Abstract

Feline injection-site sarcomas (FISS) are malignant skin tumours of mesenchymal origin, the treatment of which is a challenge for veterinary surgeons. The role of surgery, radiotherapy and chemotherapy in FISS treatment has been studied, and a correlation between “clean” surgical margins and disease-free survival has been shown. In addition, clean surgical margins are one of the most important factors for achieving a low recurrence rate. The most effective method of FISS treatment includes combining radical surgery with pre- or postoperative radiotherapy. Chemotherapy may be used as a palliative method of treatment or may be considered an adjunctive therapy for surgery and radiotherapy. In cats with FISS without metastasis, the use of immunostimulant treatment with Oncept IL-2, intended as a complementary immunotherapy in association with surgery and brachytherapy, may also be considered to reduce the risk of relapse and increase the time to relapse. Additionally, this review focuses on recent advances in FISS treatment, including the use of novel compounds, such as doxorubicin conjugated to glutathione-stabilized gold nanoparticles, liposomal doxorubicin or tyrosine kinase inhibitors.

## Background

**Fig. 1:**
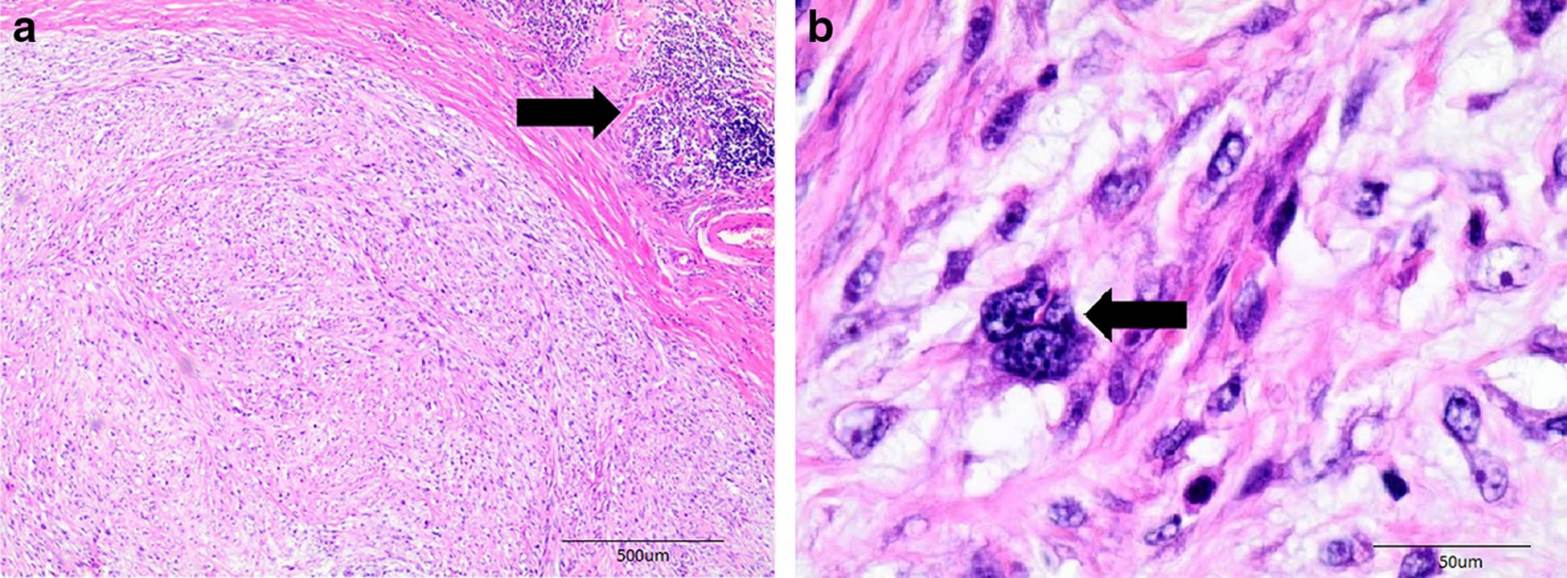
Photomicrographs of feline injection-site sarcoma. **a** An inflammatory response (*arrow*) in the peripheral part of the tumour. Haematoxylin and eosin, *bar* 500 μm. **b** A multinucleated giant cell (*arrow*). Haematoxylin and eosin, bar 50 μm

Feline injection-site sarcomas (FISS) are malignant skin tumours of mesenchymal origin that develop in 1–10 of every 10,000 vaccinated cats. The pathogenesis of this disease is unknown. However, the most accepted hypothesis is that a local post-vaccination (or injection) inflammatory process leads to neoplastic transformation [1]. This hypothesis is supported by histological findings of a central area of necrosis and the presence of inflammatory cells (Fig. 1a), including multinucleated giant cells (Fig. 1b), which have phagocytized a greyish-blue material consistent with the aluminium-based vaccine adjuvant in some cases [2–6].

FISS appear most frequently at the site of vaccinations, especially when vaccines against feline leukaemia virus (FeLV) or rabies have been used [4, 7–10], but the tumour may also develop after injection of various pharmaceutical substances, such as steroidal and non-steroidal anti-inflammatory drugs [8, 11, 12], lufenuron [13] and antibiotics. FISS may also develop after microchip implantation [14, 15] or as a reaction to non-absorbable surgical stitches [16]. FISS treatment is challenging because the local tumour recurrence rate ranges from 14 to 69% [17–20].

## Search strategy

This review was based on a search of the PubMed database (http://www.ncbi.nlm.nih.gov/pubmed) using the terms “feline injection-site sarcoma” OR “feline postvaccinal sarcoma” OR “feline vaccine-associated sarcoma” AND “treatment” OR “therapy”. Only papers written in English were included. The abstracts of the obtained hits were evaluated, and the most relevant articles were selected based on our experience in FISS. This systematic review is a synthesis of current knowledge in the field and highlights possible novel methods of FISS treatment that warrant further investigation.

### Clinical staging for treatment decision

The first-line therapy for FISS is aggressive radical surgery. Implementing adjunctive therapies, such as radiation, chemotherapy or immunotherapy, depends on the clinical status of the patient. Initially, computed tomography (CT) or magnetic resonance imaging (MRI) should be performed to determine the actual tumour size because some studies have shown that the tumour may be much larger than estimated by physical examination [21, 22]. The staging of the tumour requires a complete blood count, a serum biochemical panel, urinalysis, 3-view thoracic radiography, lymph node examination by palpation, and ultrasonography of the abdominal cavity and cytology when applicable [23]. Thoracic radiography is performed to exclude metastases to the lungs, which occur in 10–24% of FISS cases. CT has proven useful not only in planning peripheral excision margins [16, 21, 24] but also for the patient following surgical excision because it can provide information about the area that needs to be re-excised or included in the radiation treatment field [25].

### Surgery alone

Aggressive, radical surgery or wide excision is recommended. In practice, this means surgery with at least 3 cm (preferably 5 cm) margins peripherally and one fascial plane deep into the tumour. If the tumour involves adjacent bone structures, such as the scapula, a spinous process or the pelvis, bone structures must be removed during surgery [23]. Marginal excision is rarely curative and often leads to local recurrence and is therefore not recommended. It has been shown that cats subjected to aggressive excision in the first surgery have longer tumour-free intervals than cats with marginal excision (325 days versus 79 days) [26]. It was also shown by Kaplan–Meier’s product limit method that there is a significant difference in time to the first recurrence between cats treated with wide excision surgery compared to marginal excision surgery (419 days versus 66 days) [27]. After tumour excision, histopathology of the surgical margins is mandatory to ensure complete excision of the tumour.

Completeness of surgical margins is considered the most important predictive factor for FISS treatment. In a study by Cronin et al. [22], five cats with tumour cells in the margins of the resected tissue showed a median disease-free interval (DFI) of 170 days, while 26 cats with negative tumour margins showed a median DFI of 700 days (P < 0.0001). Using post-surgery 3D histological margin status evaluation, Giudice et al. [19] demonstrated that tumours with infiltrated margins recurred approximately 10 times more frequently than those with non-infiltrated margins (P = 0.001). The tumour should be resected with at least a 5 cm margin surrounding the palpable tumour edge because it prolongs the disease-free survival time from 499 to 1461 days [20]. It has also been shown that disease-free survival can be prolonged if the surgery is performed by an experienced surgeon from a referral veterinary clinic. The median time to first recurrence was found to be 274 days, compared to 64 days when the surgery was performed at a non-referral institution [27].

The owner should also be informed about the post-surgical complications that may appear. Minor complications, such as chronic constipation and pneumonia, are managed successfully by medical treatment in most cases. Major complications, such as dehiscence of the surgical incisions, e.g., when the tumour is localized in the interscapular region or laryngeal paralysis, requires a second surgical intervention and intensive care treatment.

It must be emphasized that not all tumours can be completely removed surgically, possibly due to significant tumour size or localization in the intra-scapular region with infiltration into the thoracic part of the spine or due to the poor general condition of the animal, such as phase 3 or 4 chronic renal failure.

### Adjuvant radiation

Even radical surgery may be insufficient (the recurrence rate is up to 70%, and the disease-free survival time is approximately only 6 months [4]); therefore, radiotherapy is often used pre- or postoperatively. When radiotherapy is administered prior to surgery, metastatic seeding during surgery is less likely. Kobayashi et al. [28] suggest using pre-operative radiation (using cobalt photons, 3.0 Gy in 16 daily fractions) for cats with FISS for long-term tumour control, especially in cases where tumour cells at the margin of the resected specimen are not identified. Unfortunately, these authors did not assess the influence of pre-operative irradiation on the overall survival of the cats.

On the other hand, Mayer et al. [29] have claimed, based on analysis of the medical history of 76 cats with FISS, that curative radiation therapy in combination with surgery results in long-term survival. These researchers examined the overall survival of cats after pre-operative radiation and postoperative radiation, and they showed that significantly longer overall survival was obtained for cats with postoperative radiation (705 days versus 310 days). However, they maintained that the method of selection for cats for pre-operative radiation may have biased their results.

Eckstein, in his retrospective analysis of radiation therapy in 76 cats with FISS [30], claimed that both post-surgical curative radiation (12 × 4.0 Gy delivered on a Monday–Tuesday–Thursday–Friday schedule or 12 × 5.0 Gy delivered on a Monday–Wednesday–Friday schedule) and coarse fractionated radiotherapy (4 fractions of 8.0 Gy each administered once a week) were effective.

Cronin et al. [22] performed a study on 33 cats with histopathologically confirmed FISS treated with radiation therapy followed by surgery. They showed that obtaining only a negative tumour margin significantly (P < 0.001) prolonged DFI (median DFI of 700 days versus 112 days in cats with tumour cells at the margin). All other factors, such as tumour volumes, number of prior tumour excisions and various descriptors of the radiation therapy techniques, played no role in prolonging DFI.

Cohen et al. [31] analysed the medical records of 50 cats with FISS that underwent surgery and electron beam radiation (13 fractions of 400 Gy, given each Monday, Wednesday and Friday); in contrast to the results obtained by Cronin et al. [22], they identified an association between tumour size, the number of prior tumour excisions, median survival time or DFI. Moreover, Cohen et al. [31] showed that the size of tumours before the first surgery was correlated with the median survival time (smaller tumours had longer survival times). In addition, DFI and survival time decreased as the time between surgery and the start of radiotherapy increased. Moreover, the time between the onset of the disease and the initiation of the radiotherapy influenced the survival time, as cats that started radiotherapy earlier showed a longer survival time. On the other hand, while the number of surgical procedures did not influence the median survival time, this factor was crucial for DFI; cats that underwent only one surgery had a longer DFI than cats with more than one surgery (median DFI 469 days and 345 days, respectively). The 1-year and 2-year survival rates were 86 and 44%, respectively.

Nolan et al. [32] analysed the medical records of cats with FISS (each cat had complete local and systemic staging, including CT-based planning) that underwent surgery together with stereotactic body radiation therapy (SBRT) (ranging from 24 to 35 Gy delivered in 3 to 5 fractions either on consecutive or alternative days). These authors concluded that SBRT may be used as a palliative treatment or may be considered for use prior to surgery to downstage the disease. However, their study had some limitations, such as a small population of heterogeneous patients (11 cats), various radiation doses and protocols as well as the lack of a control group of additional anticancer treatments after SBRT.

Despite the fact that radiotherapy combined with radical surgery seems to be the most efficient therapy for FISS, the short- and long-term side effects of radiotherapy should be considered prior to the decision to administer treatment. The short-term effects of radiation that appear within a few weeks after the completion of a course of fractionated radiotherapy include dermatological changes (e.g., skin erythema, mucositis) and gastrointestinal tract disorders (e.g., vomiting, diarrhoea) [33]. Some of the early side effects may also be iatrogenic due to inappropriately performed radiation, e.g., skin necrosis due to prolonged exposure or excessive radiation dose. Radiotherapy should therefore be performed only by qualified veterinary surgeons. Although rarely described in veterinary medicine, long-term radiation toxicity effects, such as fibrosis, atrophy, vascular damage, neural damage and a range of endocrine and growth-related effects, are well documented in human medicine [33]. Because these long-term side effects appear mostly within months to years after radiation, the lack of information about them in cats with FISS is likely due to a lack of studies on the long-term monitoring of the health status of cats or their survival times being too short. Thus, studies monitoring the long-term health conditions of cats with FISS that received radiotherapy are needed. Minimal side effects were observed after brachytherapy and SBRT, which indicates that these represent the safest methods of radiotherapy.

### Role of chemotherapy in combined treatment

Chemotherapy should not be used as monotherapy, although it may be considered a palliative treatment or in an adjuvant or neoadjuvant setting in a multimodal approach. Cytostatic drugs that have been used in FISS treatment include doxorubicin, ifosfamide, carboplatin, cyclophosphamide, mitoxantrone and vincristine. However, the effectiveness of chemotherapy in the treatment of FISS remains debatable. One of the most significant issues concerning the interpretation of results from clinical trials assessing the effectiveness of chemotherapy for FISS treatment is that these studies were performed on a very small number of cats. The results of a phase II clinical trial of FISS treatment with ifosfamide (900 mg/m^2^ body surface area IV, administered twice with a 3-week interval), showing that 11 of 27 cats showed a complete or partial response, suggest that this drug may serve as an efficient adjuvant treatment [34].

### Doxorubicin in multimodal FISS treatment

The results of studies performed in the USA on 71 cats after partial excision of FISS and radiotherapy show a markedly longer remission time in patients that were additionally treated with doxorubicin administered between three to five times at 3-week intervals than in patients with no doxorubicin therapy. Furthermore, the study indicated a definite prolonged remission time in patients treated with doxorubicin, which provided a better quality of life for those patients [35]. Administration of doxorubicin did not, however, prolong the disease-free survival time.

Moreover, when comparing the results of a randomized, multicentre study on 108 cats with FISS treated with either doxorubicin or liposomal doxorubicin (Caelyx) to a historical control group of cats with FISS treated with surgery alone, the cats receiving chemotherapy showed prolonged DFI (388 versus 93 days) [36]. In a pilot study including 10 cats with advanced FISS, it was shown that liposomal doxorubicin used with palliative daily fraction radiotherapy could also be used as a depot radiosensitizer [37]. Additionally, in a study performed on 12 cats with non-resectable FISS, combining doxorubicin with cyclophosphamide resulted in a partial response in 50% of cats and significant improvement in the survival time of responders [38]. Moreover, it has been demonstrated that doxorubicin used in neoadjuvant chemotherapy combined with anatomical resection of the tumour and muscle compartment prolongs both DFI and the tumour-free survival time [39].

A few studies have shown that adding doxorubicin treatment to surgery and radiotherapy or postoperative electron beam therapy has no influence on disease-free survival time, lifetime and tumour remission [17, 31, 40]. However, those studies included small numbers of animals and have low power. As a result, doxorubicin is considered to be the drug of choice for FISS treatment if a patient is classified for adjuvant chemotherapy.

The main impediments to doxorubicin administration, however, are the side effects related to its poor biodistribution and high toxicity. Doxorubicin has a short drug half-life and it is taken up by the mononuclear phagocyte system as quickly as 5 min after intra-venous administration, which leads to a lower drug concentration in the tumour. Moreover, doxorubicin administration in cats causes anaemia, myelosuppression and nephrotoxicity [36]. As a result, cats with diseases such as chronic renal failure, haemolytic anaemia, autoimmune-mediated anaemia or various bone marrow disorders are excluded from doxorubicin administration. New substances, which will overcome these limitations, are under investigation.

### Preclinical trials

In vitro studies have shown good cytotoxic effects of doxorubicin, vincristine, mitoxantrone and paclitaxel in FISS cell lines [41, 42]. However, the results of in vitro studies do not always correlate with those of in vivo studies.

Masitinib, which is a selective and efficient tyrosine kinase inhibitor due to binding at c-Kit, PDGFR and Lyn, has shown an anti-proliferative effect in two FISS cell lines [43]. Nevertheless, in further studies, when it was used together with radiotherapy, it has not enhanced sensitivity to radiation in FISS cells [44]. Further clinical studies to assess Masitinib effectiveness are needed, although studies on the other tyrosine kinase inhibitor Toceranib, which was registered for canine mast cell tumour treatment, on 18 cats failed to show efficacy for FISS treatment [45].

Liposomal doxorubicin (Caelyx), both in vitro and in vivo using a xenograft murine model, was found to be efficient in combination with radiotherapy for adjuvant treatment of FISS. Animals that received combined therapy (Caelyx 3 mg/kg and radiation 2 × 3.5 Gy or Caelyx 3 mg/kg and radiation 2 × 5 Gy) showed significantly smaller tumour sizes than the control groups [46].

Our results demonstrated that glutathione-stabilized gold nanoparticles non-covalently modified with doxorubicin (Au-GSH-Dox) could enhance the anti-tumour efficacy of free doxorubicin in FISS cell lines with high P-glycoprotein activity (the main efflux pump responsible for multidrug resistance and one of the reasons for ineffective chemotherapy treatment) [47]. The results of in vitro studies confirmed by *in ovo* studies were significant (P < 0.001), and the reduction in tumour size was visible after a single intra-tumoural injection of Au-GSH-Dox [48]. Furthermore, a chick embryo chorioallantoic membrane (CAM) model was established by our group to expand knowledge of the biological behaviour of FISS and to screen novel anticancer agents [48, 49].

However, to confirm the efficacy of Caelyx, Au-GSH-Dox or Masitinib, further in vivo studies should be performed.

### Immunotherapy

A recombinant canarypox virus (ALVAC) expressing feline interleukin-2 (IL-2) (an important T cell stimulatory cytokine approved as an exogenous anti-tumour agent) is approved for use as adjunctive immunotherapy after surgery and brachytherapy for primary FISS in cats without enlarged lymph nodes and lung metastasis. This treatment should be administered in five subcutaneous injections in the region of the sutures after tumour excision, which may cause some controversy due to the pathogenesis of the disease. However, the efficiency and safety of ALVAC (6 consecutive doses of less than 10^6.5^ EAID_50_ ALVAC IL-2 in the low-dose group and up to 10^8.9^ EAID_50_ ALVAC IL-2 in the high-dose group, which was initially given 6 times once a week and then every 2 weeks (e.g., days 0, 7, 14, 21, 35, and 49), with the first dose administered on the day before radiotherapy as an adjunct therapy for surgery and brachytherapy (4 fractions at 6.5 Gy on day 1, day 1 + 8 h, day 3, and day 3 + 8 h), has been demonstrated over a 1-year follow-up period. In a study performed on 71 cats with primary FISS, the authors demonstrated one of the highest DFI values (>765 days) reported thus far in FISS [50]. This veterinary medicinal product (Oncept IL-2, Merial) has been successfully registered in most European countries.

## Conclusions

The selection of treatment for FISS depends on the clinical staging of the disease. Performing CT or MRI is recommended as an additional diagnostic tool to assess the actual size of the tumour and exclude metastasis. The most effective method of FISS treatment includes combining radical surgery with pre- or postoperative radiotherapy. Histological margin determination is the most important predictive factor for FISS, and resecting tumours with clear margins significantly prolongs DFI. Chemotherapy may be used as a palliative method of treatment or may be considered, depending on the condition of the animal, as adjunctive therapy for surgery and radiotherapy. Doxorubicin seems to be the drug of choice, although there remains insufficient evidence regarding its efficacy. In cats with FISS without metastasis, the use of immunostimulant treatment with Oncept IL-2 as a complementary immunotherapy, in association with surgery and brachytherapy, may also be considered to reduce the risk of relapse and increase the time to relapse. However, this vaccine is not a classical vaccine and is not intended for prophylactic use or to stimulate specific active immunity.

Good co-operation between first-stage veterinary doctors, veterinary oncologists, veterinary surgeons from reference clinics and histopathologists, who assess for clear margins (the main prognostic factor), is essential for complete management of cats with FISS.

## Abbreviations

ALVAC: recombinant canarypox virus
Au-GSH-Dox: glutathione-stabilized gold nanoparticles conjugated to doxorubicin
CAM: chorioallantoic membrane
CT: computed tomography
DFI: disease-free interval
FeLV: feline leukaemia virus
FISS: feline injection-site sarcoma
IL-2: interleukin-2
IV: intravenously
MRI: magnetic resonance imaging
SBRT: stereotactic body radiation therapy
